# The Comparison Between Different Hospital Market Definition Approaches: An Empirical Analysis of 11 Representative Diseases in Sichuan Province, China

**DOI:** 10.3389/fpubh.2021.721504

**Published:** 2021-08-18

**Authors:** Liyong Lu, Ting Chen, Tianjao Lan, Jay Pan

**Affiliations:** ^1^West China School of Public Health and West China Fourth Hospital, Sichuan University, Chengdu, China; ^2^Institute for Healthy Cities and West China Research Center for Rural Health Development, Sichuan University, Chengdu, China

**Keywords:** hospital market definition approaches, hospital competition, comparison, representative diseases, China

## Abstract

**Objective:** This study aims to provide empirical evidence for the controversy about whether the inference is consistent if alternative hospital market definition methods are employed, and for which definition method is the best alternative to the predicted patient flow approach.

**Data sources:** Collecting data from the discharge data of inpatients and hospital administrative data of Sichuan province in China in the fourth quarter of 2018.

**Study Design:** We employed Herfindahl–Hirschman Index (HHI) as the proxy of market competition used as an example to measure the hospital market structure. Correlation coefficients of HHIs based on different definition methods were assessed. The corresponding coefficient of each HHI estimated in identical regression models was then compared. In addition, since the predicted patient flow method has been argued by the literature of its advantages compared with the previous approaches, we took the predicted patient flow as a reference to compare with the other approaches.

**Data Extraction Methods:** We selected the common diseases with a significant burden, and 11 diseases were included (902,767 hospitalizations).

**Principal Findings:** The correlation coefficients of HHIs based on different market definition methods are all significantly greater than 0, and the coefficients of HHIs are different in identical regression models. Taking the predicted patient flow approach as a reference, we found that the correlation coefficients between HHIs based on fixed radius and predicted patient flow approach is larger than others, and their parameter estimates are all consistent.

**Conclusion:** Although the HHIs based on different definition methods are significantly and positively correlated, the inferences about the effectiveness of market structure would be inconsistent when alternative market definition methods are employed. The fixed radius would be the best alternative when researchers want to use the predicted patient flow method to define the hospital market but are hindered by the data limitations and computational complexity.

## Introduction

Several empirical hospital market definition approaches were proposed in previous studies, including the geopolitical boundaries, fixed radius, variable radius, actual patient flow, and predicted patient flow (1–5). The hospital market structure could be further measured based on the defined market, such as hospital market concentration or competition, privatization rate, and the market size. The structure-conduct-performance (SCP) paradigm hypothesizes a causal link from the market structure to the conduct of the hospital and then to the industry performance, mainly reflected in the medical quality and cost (6–8). An extensive empirical literature about the effectiveness of hospital market structure was carried out (6, 7, 9–15). However, these empirical studies employed different market definition methods, which would affect the empirical results (16, 17). Considerable debate exists about how big the difference is in the structure measurement of the hospital market defined by different methods, and whether the inference is consistent if alternative hospital market definition methods are employed. The empirical evidence is limited. This study aims to compare the structure measurement of hospital markets defined by different methods and to provide preliminary instructions on how to choose the definition method.

As the utilization of hospital competition has been increasingly addressed as an essential strategy to improve the efficiency of the healthcare delivery system (18–24), a series of empirical literature examined the effects of hospital competition on hospital medical services supply. However, the findings of these empirical studies are inconsistent (6, 7, 16, 25). We selected hospital market competition as the measurement of the hospital market structure in this study to compare the different market definition methods.

Two previous empirical studies^1^ have selected the hospital competition to measure the market structure and compared the competition degree of the hospital market defined by different market definition methods. Garnick et al. (17) took community hospitals in California as the study objects. Using the number of competitors of the market defined by geopolitical boundaries, the distance between hospitals (i.e., fixed radius), and patient-origin data (i.e., actual patient flow) as the independent variable, and the average cost per admission and the presence of an obstetrics unit as dependent variables, Garnick et al. (17) compared the correspondence parameter estimates through multivariate regression analysis. They found that the results are similar using either the fixed radius (15 miles radius) or county market areas, while the patient-flow-based variables give different estimated results. Based on the community hospitals in the U.S., Wong et al. (16) compared the competition degree of the hospital market defined by different definition methods (including geopolitical boundaries, fixed radius, variable radius, and actual patient flow). They found that the competition of the hospital market defined by different methods is significantly and positively correlated by comparing the correlation coefficient between HHIs based on different definition methods. They also used each HHI, as an explanatory variable, independently in identical hospital cost function regressions, and then compared the corresponding parameter estimates, finding that the inferences about the effect of competition on hospital costs remain the same when alternative hospital competition measures are employed.

However, it is noteworthy that both studies were based on the US, the largest developed country in the world, which would be quite different from a developing country, such as China (the largest developing country) in terms of many aspects, such as social and economic development, as well as healthcare system settings. Due to the structural differences between China and the United States, it is unknown whether the evidence based on the United States can be applied to China. For example, a certain type of hospital (community hospital) was selected as the study object in both studies. In China, competition pressure exists among hospitals of different types and levels, leading to the findings of previous studies that selected a certain type of hospital as the study object cannot be applied to China. The referral system is incomplete in China; patients can choose their preferred hospitals without obtaining referrals from the primary healthcare institutions in advance (29), leading to competition among different levels of hospitals.

To our knowledge, limited studies took into account the heterogeneity among diseases into the hospital market definition. Patients often select hospitals according to their disease types, leading to the distinctness of the hospital market for different diseases (30). To consider the different features across diseases, we focused on 11 representative diseases to define their market, respectively, and further compared the competition impacts based on different hospital market definitions.

In addition to comparing the geopolitical boundaries, fixed radius, variable radius, and actual patient flow (16, 17), we further included the predicted patient flow method proposed by Kessler and Mcclellan (5) in our study, which has been argued by the literature of its advantages compared with the previous approaches (5, 11, 31). However, whether structure measurement of the hospital market defined by the predicted patient flow approach is different from other methods has not yet been studied. Our study would contribute to the literature by adding new evidence.

We selected the representative common disease with significant economic burden based on the inpatient volume and medical costs according to the International Statistical Classification of Diseases and Related Health Problems, 10th Revision (ICD-10), a total of 11 types. After defining the hospital market with the geopolitical boundaries, fixed radius, variable radius, actual patient flow, and predicted patient flow, we calculated the Herfindahl–Hirschman Index (HHI) to measure the hospital competition degree. Correlation coefficients of HHIs based on different definition methods were assessed. In the corresponding coefficient of each HHI estimated in the identical regression models, each HHI was independently used as an independent variable and inpatient costs as the dependent variable were then compared. The main results show that although the HHIs based on different hospital market definition methods are significantly and positively correlated, their coefficients are different in identical regression models, which means that the inference about the effectiveness of market structure would be inconsistent when alternative definition methods are employed. In addition, taking the predicted patient flow as a reference, the results show that the correlation coefficient between HHIs based on fixed radius and predicted patient flow approach is larger than others, and their estimated coefficient is consistent in the direction for all selected diseases, suggesting that when researchers want to use the predicted patient flow method to define the hospital market, but are hindered by data limitations and the challenge in computation, the fixed radius would be a good alternative. We also initially discussed how to choose definition methods to provide preliminary instruction for empirical studies when it is necessary to choose definition methods.

This study makes the following contributions to the literature: (1) This paper aims to bridge the gap in the literature by comparing the structure measurement of the hospital market defined by different definition methods in China. To the best of our knowledge, this is the first attempt to compare the structure measurement of the hospital market defined by different definition methods in developing countries and the first to compare the predicted patient flow approach with other definition methods. (2) This study provides empirical evidence and preliminary instruction for researchers when they need to select definition methods to define the hospital market.

This paper is organized as follows: Section Background displays briefly the background of the Chinese hospital provision system; Section Market Definition Approach overviews the principle of the hospital market definition approaches; Section Materials and Methods gives a description of materials and methods, including setting, data source, the principle of disease selecting, the details of different definition methods to define the hospital market, and the methods of statistical analysis; Section Results outlines the empirical results; Section Discussion summarizes the discussion.

## Background

Public hospitals, as the key providers in the Chinese health system, were run and financed by the government through financial subsidies and government-organized insurance programs during the period of the old communist planned economy (32). In 1980, the marketization reform was launched by the Chinese government. The financial support of the government toward hospitals was reduced drastically in this reform, and most insurance programs were either dismantled or significantly weakened (33). On average, only 10% of the revenue of the hospital comes from government subsidies, and the vast shortfall needs to be filled by patient fees (15). Hospitals are allowed to earn revenues from the sales of drugs and high-tech service charges to maintain the daily operation activities. The prices of drugs and advanced diagnostic tests, such as CT and MRI scans, were allowed to make up from 10% to 15% (34), leading hospitals to have great incentive to either over-prescribe or diagnosis.

The primary healthcare facilities are significantly underutilized partly because the patients do not trust the quality of their services (35). Combined with the absence of a functioning gate-keeping and referral system, patients are tended to visit high-level hospitals instead of low-level hospitals, even for minor conditions (36), leading to patients flooding into high-level or big hospitals. Since the majority of the revenue of the hospitals comes from the fees of the patients, the hospitals want to retain the patients rather than referring them elsewhere (37).

In 2009, the Chinese government launched a new round of national health reforms to solve the problem of “*Kan Bing Nan, Kan Bing Gui*” (getting medical care is difficult and expensive) (38). Pro-competition policies aiming to improve the hospital efficiency were implemented followed by the reform (39), including separating government regulatory and operational control of public hospitals to enhance the hospital autonomy (40), relaxing the entry barriers of private hospitals, and encouraging private investment into hospital market to prompt the development of the private hospitals (41, 42), as well as transitioning the health fiscal system toward demand-side financing (national basic health insurance system) from the supply-side financing model (public providers) (28, 43, 44).

With the implementation of pro-competition policies, the number of hospitals keeps increasing dramatically, intensifying the competition degree of the hospital market. For example, the number of hospitals (including public and private hospitals) increases from 20,291 in 2009 to 33,009 in 2018. Among them, the private hospital increases more obviously, from 6,240 in 2009 to 20,977 in 2018. Jiang and Jay (45) used administrative data of all the hospitals in Sichuan Province during the period from 2002 to 2017 to analyze the trends in the development of hospital market structure from market size, market share, and market concentration (45). They found that the market competition in the hospitals has become more intense.

The patients in China could freely choose hospitals because of the absence of a functioning gate-keeping and referral system (36), leading to the hospitals having the opportunity to attract patients in the market (in other words, the patients also have the opportunity to choose hospitals). Many medical services provided by hospitals in a market generally are substitutes for each other; patients can select different hospitals for the same kind of services. Since the major revenue comes from patient fees, hospitals have great incentives to attract patients to earn more revenues. Improving the quality or reducing the cost of the services is usually the common method used by hospitals to attract patients (6). For example, hospitals would purchase high-tech medical facilities and introduce high-educated physicians as tactics for demonstrating their excellent ability for handling complicated clinical tests and treatments to attract patients (5, 46, 47). Hospitals would also decrease the medical costs of the patients by some measures, such as improving the efficiency by reducing the length of stay of the inpatients or decreasing unnecessary services to attract patients (28).

With the implementation of pro-competition policies in China, the question of the effectiveness of hospital competition has become a hot topic that received considerable attention in recent years (11, 28, 43, 48, 49). However, there is no study to systematically compare the different market definition approaches and test whether the inference is consistent if alternative definition methods are employed based on the Chinese health system. This study compares the competition of hospital markets defined by different methods and provides preliminary instructions for the research on the effectiveness of hospital competition in China to choose the market definition methods.

## Market Definition Approach

Before overviewing the principle of the hospital market definition approaches, we need to select the indicators to measure market competition degree. The number of hospitals, M-concentration ratio (M-CR)^2^, and HHI are often used to measure the market competition degree (16). However, the index of the hospital number does not reflect the market share of each hospital in the market, and the M-CR does not fully reflect the market share of every hospital in the market. Compared with these two indicators, HHI is more reasonable. The related studies commonly employed HHI to measure the hospital market competition degree (6, 16, 25). We also used HHI to measure the hospital market competition degree in this study^3^.

The empirical definition methods include the geopolitical boundaries, fixed radius, variable radius, actual patient flow, and predicted patient flow approach (50, 51)^4^. Table 1 displays the overviews of the comparison between different empirical definition approaches. We described the principle of the hospital market definition approaches as follows.

**Table 1:** The comparison between different hospital market definition approaches.

	**Geopolitical boundaries approach**	**Fixed radius approach**	**Variable radius approach**	**Actual patient flow approach**	**Predicted patient flow approach**
Whether to include potential competitors	Yes	Yes	No	No	Yes
Does it cause endogenous problems	Yes	Yes	Yes	Yes	No
Whether every hospital is assigned a unique market area	No	Yes	Yes	Yes	Yes
Whether variations of catchment areas of different hospitals are considered	No	No	Yes	Yes	Yes
Computational complexity	Low	Low	Medium	Medium	High
Data requirements	Low	Low	High	High	High

### Geopolitical Boundaries Approach

The administrative units, such as the county or metropolitan statistical areas (MSAs) are generally used in the geopolitical boundaries approach to define the hospital market (25). The hospital market defined by this method includes all competitors in an administrative unit. Computational ease and low data requirements are the main advantages of this definition method. In addition, this definition method can capture the potential competitors and is compatible with socio-economic data that are frequently made available at the administrative units, such as county and MSA (16, 17).

The disadvantage of this method is that the administrative units cannot accurately reflect the area of the market. The market area would be overestimated if the high-level administrative unit is used, otherwise, underestimated. For example, when the administrative unit of a province is adopted as the geopolitical boundary to define the hospital market, hospitals located in the province are regarded as the same market. In fact, hospitals in a province that are geographically far apart are usually not in the same market, so the market would be overestimated. The hospitals in a county often compete with the hospitals in the neighboring counties. If the administrative unit of a county is used as the geopolitical boundary, the market would be underestimated. In addition, since the hospitals located at the administrative unit boundary often compete with the hospitals located just outside of the boundary, the market of these hospitals defined by this method would be inaccurate. Moreover, the measures of competition for all of the hospitals in a market (i.e., an administrative unit) are all identical. However, hospitals usually face competition at different levels of intensity (9, 16).

### Fixed Radius Approach

Luft and Maerki (2) proposed the fixed radius approach to defining the hospital market. This method uses the analyzed hospital as the center of the circle, the predetermined length as the radius, and then makes the circle to define the hospital market (2). Luft et al. (52) analyzed the hospitals in urbanized areas of California and found that the radius length of 15 miles can cover over 90% of the patients of the hospitals, then the 15 miles was often used as the radius length to define the hospital market (16, 25).

Every hospital is assigned a unique market area under the fixed radius approach. In addition, the fixed radius approach could avoid some of the drawbacks of the geopolitical boundaries approach. For example, for the hospital located at the administrative unit boundary, its market defined by the fixed radius approach can include the hospitals in the neighboring administrative units. However, the fixed radius length is the major limitation of this method (3). It is unreasonable to use the same radius length for different types or levels of hospitals. For instance, the market area of tertiary hospitals would be larger than primary hospitals, but the difference has not been reflected by the fixed radius approach. In addition, the shape of the hospital market defined by this method is the circle, which would not meet the reality.

### Variable Radius Approach

Phibbs and Robinson (4) proposed a market definition method that allows the radius length to be different for distinct hospitals. This method was improved by Gresenz et al. (53). Based on the fixed radius approach, this method allows for different radius lengths for different hospitals. When calculating the market radius length of a hospital, it is required that the radius length could cover 90% (or 75%) of hospital patients (or discharges).

Compared with the fixed radius method, this method considers the variations in the size of catchment areas of hospitals and allows for the radius length to be distinct for different hospitals. However, due to the utilization of the actual patient flow information to calculate the different radius length, the main disadvantage of the method is in ignoring the potential competitors which could not be known by the patient revealed utilizations. In addition, just as the fixed radius method, the shape of the market defined by the variable radius method is also the circle, which would not meet the reality.

### Actual Patient Flow Approach

The actual patient flow approach does not restrict the shape of the hospital market to a circle. Generally, the hospital market defined by this method is the collection of geographic areas (such as zip codes) where most of the patients of the hospital (75 or 90%) come from Zwanziger et al. (3) and Wong et al. (16).

Like the variable radius approach, since this method is also based on the actual patient flow information to define the collection of geographic areas, the main disadvantage of this method is also in ignoring the potential competitors.

### Predicted Patient Flow Approach

The HHI based on the geopolitical boundaries, fixed radius, variable radius, and actual patient flow methods would cause endogeneity problem when it is used as a proxy of hospital competition and further as an independent variable in the regression model to analyze the effect of competition on hospital performance (such as medical costs, quality, and patient volume). For example, HHI is calculated by summing the squares of the market share of each hospital usually calculated by the medical revenue or discharge of the hospital. The medical revenue or discharge of the hospital often reflects the performance of the hospital, leading to the endogeneity problem caused by reverse causality (5, 11, 31).

Kessler and Mcclellan (5) proposed a new approach to solve this endogeneity problem, called the predicted patient flow approach. This method constructs the choice model of patients to predict the probability of patients choosing each hospital in the choice set, and then to calculate the HHI of hospitals based on the predicting probability. The exogenous variables, such as individual characteristics (i.e., gender, age, and ethnicity), hospital characteristics (i.e., hospital ownership), and the distance between the patient and the hospital, are used to predict the probability.

The hospital market defined by this method can include the potential competitors of hospitals, and avoid the endogenous problem and shortcomings of other traditional definition methods (5, 11, 16, 31, 54). Although this method has been argued by the literature of its advantages compared with the previous approaches (5, 11, 31), it is still not commonly used in related research due to the high data requirements and the complex calculation process.

## Materials and Methods

### Setting

This study is based on Sichuan, a southwestern province of China, with an area of 486,052 square kilometers. In 2018, the per capita GDP of Sichuan was 48,883 yuan, which is a medium level in China. In the same year, the residences of Sichuan are about 83.41 million, making it the fourth most populous province in China (55). It is of great practical significance to analyze a hospital market covering such a large population (28).

**Figure 1:**
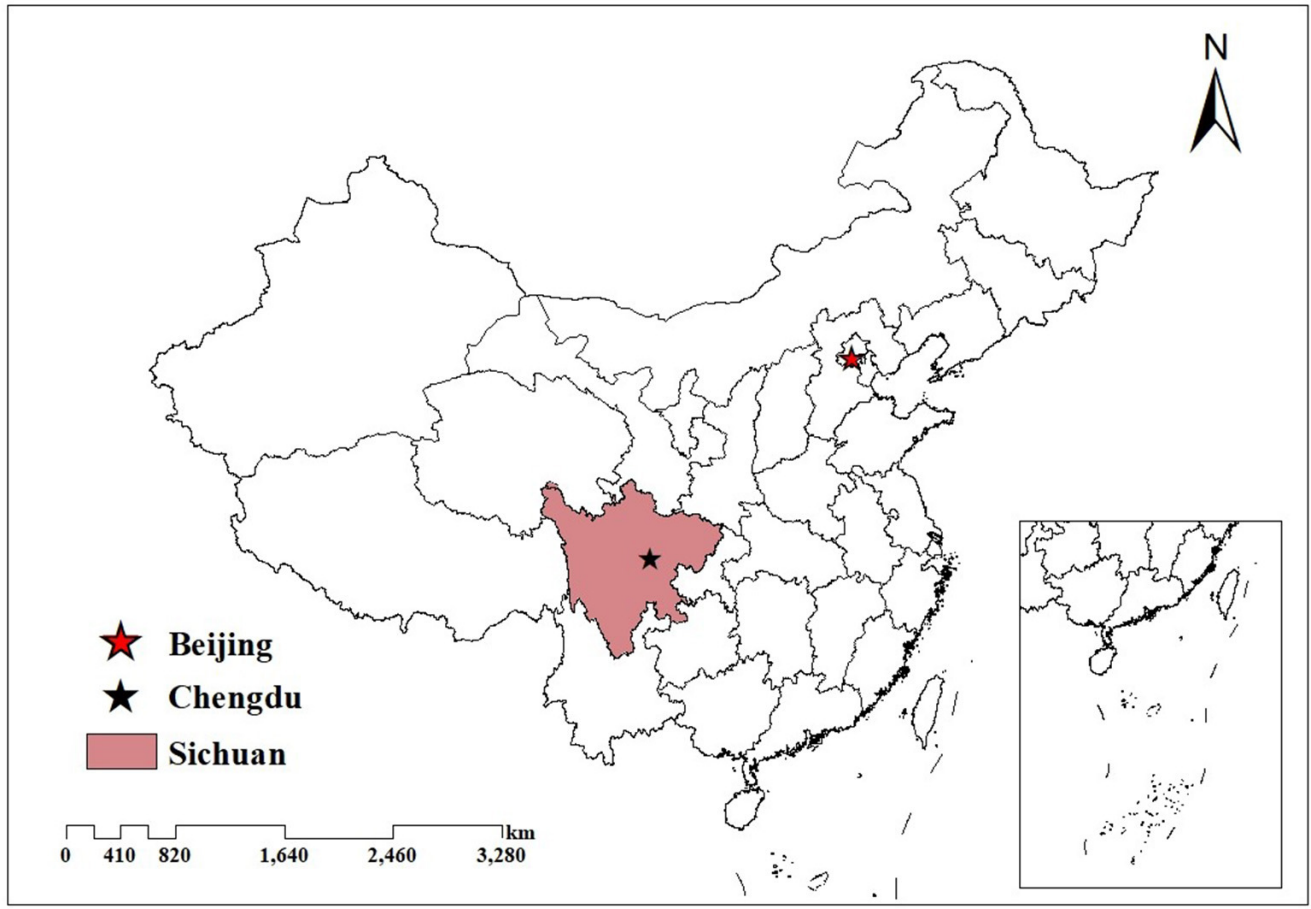
Sichuan's location in China.

As the largest developing country in the world, the topography, economy, and population distribution in China are very unbalanced, and the situation in Sichuan is similar to the whole country^5^. It is like a miniature of China, leading to the empirical findings based on Sichuan that could represent the overall situation of China (28). Figure 1 displays the location of Sichuan in China.

### Data Source

The patient-level and part of hospital-level data used in this study were collected from the discharge data of inpatient in the fourth quarter of 2018 (from September to December) managed by Sichuan Provincial Health Statistics Support System Database, including the basic information about the hospital, inpatients, admission, disease, and treatment information. The basic information of a hospital includes hospital ID, ownership, level, address, and whether profitable or general. The basic information about a patient includes the age, gender, and address of the patient. The admission information includes the urgency at admission from the emergency department, (critically urgent, urgent, or general). The disease information includes the ICD-10 code and the name of the primary and secondary diagnosis. The treatment information includes inpatient cost (total cost and sub-group cost) (28, 56). The number of beds and name of hospitals was collected from the hospital administrative data in 2018, which is reported by the hospital annually to the health commission of Sichuan province at the end of the year. Using the hospital ID, we matched the discharge data and the hospital administrative data. The latitude and longitude of the hospital and patient locations were geocoded from Gaode map (a website similar to Google maps) based on the hospital name and address, as well as the address of the patients, which were utilized to obtain the distance metrics among hospitals, and between hospitals and patients.

### Disease Selecting

Patients usually select hospitals according to the disease, which would lead to the hospital markets being distinct for different diseases (30). The defined market would be inaccurate if the heterogeneity effects among diseases on the market definition are not considered. To exclude the different features across diseases, we selected representative diseases to define their respective markets and further compared the market defined by different methods.

Based on the inpatient volume and medical expenses, we selected the common diseases with a significant economic burden^6^. We calculated the total inpatient volume and total inpatient cost for each disease and then included the top 20 diseases in terms of both the total inpatient volume and total inpatient cost, including chronic obstructive pulmonary disease, pneumonia, intervertebral disc disease, cerebral infarction, chronic ischemic heart disease, spinal ankylosis, cholelithiasis, senile cataract, schizophrenia, non-insulin-dependent diabetes, and obstructive and reflux uropathy. Ultimately, 11 diseases and 902,767 hospitalizations were included (about 30% of total hospitalizations).

We cleaned up the data of 11 selected diseases before analysis. Table 2 shows the details of the data cleaning process^7^.

**Table 2:** The details of disease selection and data cleaning.

**Disease**	**ICD-10**	**Criteria**			
		**Including all inpatients of this type of disease admitted to hospitals in Sichuan province in the fourth quarter of 2018**	**Excluding those individuals whose address are missing**	**Excluding those individuals whose medical costs are missing**	**Final data: number of inpatients**
Non-insulin dependent diabetes	E11	42,923	141	12	42,770
Schizophrenia	F20	47,230	193	15	47,022
Senile cataract	H25–H26	72,100	194	1	71,905
Chronic ischemic heart disease	I25	76,593	391	6	76,196
Cerebral infarction	I63	79,834	420	1	79,413
Pneumonia	J12–J18	180,191	551	21	179,619
Chronic obstructive pulmonary disease	J44	152,319	722	30	151,567
Cholelithiasis	K80	59,288	143	6	59,139
Spinal ankylosis	M47	59,490	434	28	59,028
Intervertebral disc disease	M50–M51	100,511	746	1	99,764
Obstructive and reflux uropathy	N13	36,524	177	3	36,344

### Defining Market and Measuring Competition

We used different market definition methods to define the hospital markets, respectively, based on the principle in section Market Definition Approach, and then HHI to measure the competition degree of the market.

#### Geopolitical Boundaries Approach

County was used as the geopolitical boundary to define the hospital market, that is, the hospitals in the same county were divided into the same market. HHI was calculated by summing the squares of the market share of each hospital calculated by the inpatient volume of the hospital.

(1) $$\begin{array}{l} HHI_{\text{c}}=\overset{n}{\underset{h=1}{\sum }}{\left(V_{hc}/TV_{c}\right)}^{2}=\overset{n}{\underset{h=1}{\sum }}{S_{hc}}^{2} \end{array}$$

where, *c* denotes the county, *V*_*hc*_ refers to the inpatient volume of hospital *h* in county *c*, while *TV*_*c*_ denotes the sum of the inpatient volume of all the hospitals in county *c*. S_*hc*_ is the market share of hospital *h* in county *c*, and *HHI*_*c*_ measures the competition degree of county *c*.

#### Fixed Radius Approach

According to previous studies, we selected 15 miles as the radius length (16, 17, 28, 49, 52). All hospitals in a circle are divided into the same market. Then we calculated the HHI to measure the competition intensity. *HHI* was also calculated by summing the squares of the market share of each hospital calculated by the inpatient volume of the hospital.

(2) $$\begin{array}{l} HHI_{m}=\overset{n}{\underset{h=1}{\sum }}{\left(V_{mh}/TV_{mh}\right)}^{2}=\overset{n}{\underset{h=1}{\sum }}S_{mh}^{2} \end{array}$$

where, *m* denotes the hospital market defined by the fixed radius approach.

#### Variable Radius Approach

The calculation process of this method is as follows: (1) The geographical distance from the patients to the hospitals was calculated based on the longitude and latitude of patients and hospitals. (2) For each hospital, the inpatients were arranged in ascending order according to the geographical distance to the hospital, and then the number of the inpatients was accumulated until it reached 90% of the total inpatient volume of the hospital. The geographical distance of the last accumulated inpatient to the hospital was used as the radius length of this hospital. (3) Using the radius length of each hospital, we defined the hospital market for each hospital. Then, we calculated HHI to reflect the competition intensity of the market. The HHI based on the variable radius approach is the same as Formula 2.

#### Actual Patient Flow Approach

The calculation process of the above method is as follows:

(1) Since the zip code in the address of the inpatient is incomplete in our data, the county address of the inpatient was adopted to mark the geographic area of inpatients.

(2) The number of inpatients of county *c* (*NP*_*c*_) and the number of inpatients of hospital *h* (*NP*_*h*_) were calculated, respectively. Then we calculated how many inpatients in the hospital *h* (*NP*_*ch*_) came from the county *c*. We calculated *HHI*_*c*_ to measure the competition degree of county *c* based on the above indicators. The formula is as follows:

(3) $$\begin{array}{l} HHI_{\text{c}}=\overset{n}{\underset{h=1}{\sum }}{\left(NP_{ch}/NP_{c}\right)}^{2} \end{array}$$

(3) We further calculated the *HHI*_*h*_ to reflect the competition degree faced by hospital *h*.

(4) $$\begin{array}{l} HHI_{h}=\overset{n}{\underset{c=1}{\sum }}\left[\left(NP_{ch}/NP_{h}\right)\times HHI_{c}\right] \end{array}$$

According to previous literature, county *c* was excluded from the collection of geographic areas of hospital *h* if the number of inpatients from county *c* treated in hospital *h* is <1% of the total inpatients volume from hospital *h* (16, 17). In other words, only if county *c* meets the criterion (*NP*_*ch*_*/NP*_*h*_) ≥ 1% could it be included as the geographic area of hospital *h*.

#### Predicted Patient Flow Approach

This method refers to previous studies (5, 11, 31, 54). The calculation process of the predicted patient flow approach is as follows:

(1) We constructed the model that includes the hospital choice of the inpatients and variables that are exogenous to unobserved characteristics of patients and hospitals, and assumed that patients choose the hospital that maximizes their utility. The formula is as follows:

(5) $$\begin{array}{l} U_{ij}=\overset{3}{\underset{k=1}{\sum }}\left\{\begin{array}{l} \beta_{1}^{k}\left(d_{ij}-d_{ij^{+}}^{k}\right)\times z_{j}^{k}\\ +\beta_{2}^{k}\left(d_{ij}-d_{ij^{+}}^{k}\right)\times \left(1-z_{j}^{k}\right)\\ \text{+}\beta_{3}^{k}\left(d_{ij}-d_{ij^{-}}^{k}\right)\times z_{j}^{k}+\beta_{4}^{k}\left(d_{ij}-d_{ij^{-}}^{k}\right)\times \left(1-z_{j}^{k}\right)\\ \text{+}\beta_{5}^{k}\left(female_{i}\times z_{j}^{k}\right)\\ +\beta_{6}^{k}\left(age_{i}\times z_{j}^{k}\right)\\ +\beta_{7}^{k}\left(lowseverity_{i}\times z_{j}^{k}\right)\\ +\beta_{8}^{k}\left(general_{i}\times z_{j}^{k}\right)\\ +\beta_{9}^{k}\left(emergency_{i}\times z_{j}^{k}\right)\\ +\beta_{10}^{k}\left(CCI_{i}\times z_{j}^{k}\right) \end{array}\right\}+e_{ij} \end{array}$$

Where, *U*_*ij*_ denotes the utility that inpatient *i* receives from hospital *j* ∈ *J* (*J* denotes the potential hospital choice set of the patients); The choice set *J* was restricted to the hospital chosen by the inpatients and all hospitals within 100 km (5, 11, 31); *d*_*ij*_ denotes the distance from the address of the inpatient, *i* to hospital *j*; $d_{ij+}^{k}$ and $d_{ij-}^{k}$ denote the distance to the closest hospital as a good or poor substitute for hospital *j* in terms of characteristic *k*, respectively; When *k* = 1, 2, 3, $Z_{j}^{k}$ represents whether hospital *j* is a tertiary hospital, a big hospital (defined as the actual number of hospital beds over the median bed for a specific disease), or a public hospital, respectively. The variables, *female*_*i*_ and *age*_*i*_ denote the gender and age of inpatient *i*, respectively. The variables of *low severity*_*i*_ are binary indicators of whether inpatient *i* has less than three diagnosis codes in their secondary diagnoses (then high severity as the reference group). The variables of *general*_*i*_ are binary indicators of the urgency at the admission of inpatient *i* (critically urgent or urgent as the reference group). The variable, *emergency*_*i*_ indicates whether inpatient *i* is admitted through the emergency department. The variable, *CCI*_*i*_ denotes Charlson comorbidity index (CCI) to reflect the complications of inpatient *i*, and the variable, *e*_*ij*_ indicates the disturbance items.

(2) We used the above hospital choice model to predict the probability of inpatient *i* admitted to hospital *j* in the choice set *J*.

(6) $$\begin{array}{l} {\hat{P}}_{ij}=\frac{\exp\left({û}_{ij}\right)}{\sum_{jJ}\exp\left({û}_{ij}\right)} \end{array}$$

where *û*_*ij*_ is the utility of inpatient *i* admitted to hospital *j*.

(3) We calculated the HHI of inpatient *i*.

(7) $$\begin{array}{l} HHI_{i}=\underset{jJ}{\sum }{\left({\hat{P}}_{ij}\right)}^{2} \end{array}$$

(4) We calculated of HHI of hospital *j*.

(8) $$\begin{array}{l} HHI_{i}=\frac{1}{N_{j}}\underset{iI}{\sum }\left({\hat{P}}_{ij}*HHI_{i}\right) \end{array}$$

Where, *I* refers to these inpatients who might potentially choose hospital *j*. Where, $N_{j}\;=\;\sum i\in {\hat{P}}_{ij}$ reflects the expected volume of hospital *j*.

### Statistical Analysis

#### Descriptive Analysis

Using the mean, median, SD, quartile range, maximum, and minimum values, we described the distribution of HHIs of the hospital market defined by different market definition approaches.

#### Correlation Analysis

For each disease, we calculated the Pearson's correlation coefficient matrix of HHIs of the hospital market defined by different definition approaches and made the statistical test for each correlation coefficient. The range of the correlation coefficient ranges from −1 to 1. If the correlation coefficient is >0, it means that there is a positive correlation; if the coefficient is <0, there is a negative correlation; when the correlation coefficient is equal to 0, there is no linear correlation. The values, |r| ≥ 0.7 denotes strong correlation, 0.4 < |r| < 0.7 moderate correlation, and |r| ≤ 0.4 weak correlation, respectively (57).

#### Regression Analysis

To test whether the inference about the effectiveness of the hospital market structure is consistent if alternative definition methods are employed, each HHI was independently included as an explanatory variable in identical regression models using inpatient costs as a dependent variable^8^. Their corresponding parameter estimates were then compared. The association between inpatient costs and hospital competition was analyzed using the log-linear multivariate regression model. The model is as follows:

(9) $$\begin{array}{l} log\left(Cost_{idhc}\right)=\beta_{0}+\beta_{1}HHI_{dhc}+P_{idhc}+H_{hc}+C_{c}+\varepsilon_{idhc} \end{array}$$

where, *i* denotes the patient, *d* the disease, *h* the hospital, and *c* the county; *Cost* is the explained variable, which denotes the inpatient costs. *HHI* is the key independent variable, which indicates the competition intensity (or concentration). *P* is a vector of the characteristics of the patients, including gender, age, health insurance program, the admission source, urgency at admission, and CCI. *H* is a vector of variables related to the characteristics of the hospital, including the number of beds, hospital level, ownership, whether general, and whether for-profit. *C* is the vector of county characteristics, including the health personnel per 1,000 population, GDP per capita, and the number of populations, and the proportion of the urban population (urbanization rate). The variable ε denotes the error term. We used robust standard errors to correct heteroskedasticity. The inpatient costs, the health personnel per 1,000 population, GDP per capita, and the numbers of populations were natural log-transformed before regression analysis because they are highly right-skewed.

All analyses were performed using STATA 15.0. The value, *P* < 0.05 was used to determine the statistical significance.

## Results

### Descriptive Analysis Results

Table 3 shows the result of the descriptive analysis of HHIs based on different definition methods for each disease. For different diseases, the median and the quartile range of HHIs of the hospital market defined by the same defined method is distinct, indicating that for different diseases, the structure of the hospital market defined by different methods is also different. The heterogeneity effects among diseases on the market definition cannot be ignored.

**Table 3:** Descriptive analysis of HHIs based on different definition methods of selected diseases.

**Disease**	**ICD-10**	**HHI**	**N**	**Mean**	**P50**	**SD**	**P5**	**P95**	**Quartile range**
		Geopolitical boundaries	1,188	0.406	0.38	0.191	0.157	0.774	0.229
		Fixed radius	1,188	0.128	0.103	0.148	0.019	0.393	0.107
Non-insulin dependent diabetes	E11	Variable radius	1,188	0.306	0.193	0.32	0.005	1.000	0.472
		Actual patient flow	1,188	0.249	0.234	0.116	0.085	0.478	0.132
		Predicted patient flow	1,188	0.070	0.038	0.114	0.007	0.254	0.057
		Geopolitical boundaries	228	0.822	0.933	0.21	0.396	1.000	0.362
		Fixed radius	228	0.285	0.181	0.267	0.069	0.995	0.205
Schizophrenia	F20	Variable radius	228	0.414	0.258	0.398	0.013	1.000	0.948
		Actual patient flow	228	0.566	0.551	0.195	0.275	0.895	0.294
		Predicted patient flow	228	0.161	0.116	0.124	0.052	0.434	0.115
		Geopolitical boundaries	435	0.499	0.437	0.232	0.201	1.000	0.287
		Fixed radius	435	0.180	0.148	0.168	0.046	0.506	0.118
Senile cataract	H25–H26	Variable radius	435	0.272	0.167	0.301	0.008	1.000	0.393
		Actual patient flow	435	0.265	0.242	0.113	0.131	0.468	0.124
		Predicted patient flow	435	0.085	0.057	0.115	0.017	0.288	0.047
		Geopolitical boundaries	1,499	0.292	0.255	0.165	0.101	0.588	0.178
		Fixed radius	1,499	0.084	0.056	0.119	0.016	0.261	0.056
Chronic ischemic heart disease	I25	Variable radius	1,499	0.224	0.114	0.281	0.003	1.000	0.321
		Actual patient flow	1,499	0.202	0.195	0.098	0.061	0.367	0.117
		Predicted patient flow	1,499	0.068	0.04	0.111	0.007	0.238	0.050
		Geopolitical boundaries	1,399	0.333	0.301	0.172	0.121	0.659	0.192
		Fixed radius	1,399	0.099	0.062	0.133	0.015	0.335	0.078
Cerebral infarction	I63	Variable radius	1,399	0.246	0.127	0.297	0.003	1.000	0.356
		Actual patient flow	1,399	0.221	0.202	0.102	0.072	0.400	0.129
		Predicted patient flow	1,399	0.078	0.046	0.13	0.008	0.290	0.054
		Geopolitical boundaries	1,348	0.457	0.433	0.222	0.158	0.862	0.349
		Fixed radius	1,348	0.143	0.104	0.168	0.02	0.519	0.143
Pneumonia	J12–J18	Variable radius	1,348	0.325	0.208	0.335	0.005	1.000	0.544
		Actual patient flow	1,348	0.308	0.281	0.151	0.100	0.601	0.201
		Predicted patient flow	1,348	0.110	0.057	0.160	0.006	0.419	0.108
		Geopolitical boundaries	1,614	0.261	0.228	0.172	0.072	0.608	0.177
		Fixed radius	1,614	0.082	0.040	0.141	0.007	0.339	0.054
Chronic obstructive pulmonary disease	J44	Variable radius	1,614	0.202	0.075	0.276	0.002	0.976	0.291
		Actual patient flow	1,614	0.197	0.174	0.113	0.052	0.422	0.135
		Predicted patient flow	1,614	0.077	0.045	0.127	0.008	0.279	0.052
		Geopolitical boundaries	1,254	0.416	0.391	0.18	0.189	0.806	0.236
		Fixed radius	1,254	0.128	0.083	0.168	0.023	0.493	0.084
Cholelithiasis	K80	Variable radius	1,254	0.311	0.181	0.334	0.005	1.000	0.498
		Actual patient flow	1,254	0.243	0.236	0.094	0.111	0.396	0.110
		Predicted patient flow	1,254	0.081	0.041	0.137	0.008	0.307	0.058
		Geopolitical boundaries	1,468	0.223	0.182	0.163	0.072	0.532	0.135
		Fixed radius	1,468	0.072	0.039	0.124	0.007	0.215	0.063
Spinal ankylosis	M47	Variable radius	1,468	0.184	0.076	0.259	0.002	0.899	0.228
		Actual patient flow	1,468	0.165	0.145	0.096	0.056	0.355	0.107
		Predicted patient flow	1,468	0.054	0.029	0.098	0.005	0.199	0.036
		Geopolitical boundaries	1,579	0.229	0.177	0.159	0.093	0.556	0.153
		Fixed radius	1,579	0.071	0.039	0.127	0.008	0.263	0.042
Intervertebral disc disease	M50–M51	Variable radius	1,579	0.176	0.066	0.259	0.002	1.000	0.223
		Actual patient flow	1,579	0.154	0.139	0.085	0.054	0.314	0.082
		Predicted patient flow	1,579	0.053	0.025	0.12	0.005	0.192	0.028
		Geopolitical boundaries	1,038	0.395	0.349	0.203	0.183	0.853	0.245
		Fixed radius	1,038	0.127	0.086	0.158	0.021	0.442	0.087
Obstructive and reflux uropathy	N13	Variable radius	1,038	0.305	0.183	0.336	0.005	1.000	0.486
		Actual patient flow	1,038	0.226	0.201	0.105	0.102	0.418	0.123
		Predicted patient flow	1,038	0.059	0.029	0.117	0.006	0.205	0.040

For the same disease, the median of HHI of the hospital market defined by the predicted patient flow is smaller than that of the hospital market defined by other methods, while the geopolitical boundaries approach is larger than other methods. This finding means that the average competition degree of the hospital market defined by the predicted patient flow is bigger than that of the market defined by other methods, while the geopolitical boundaries approach is smaller than the other methods.

For the same disease, the quantile range of HHI of the market defined by the variable radius approach is larger than that of the market defined by other approaches, while the predicted patient flow approach is smaller than others.

### Correlation Analysis Results

Table 4 shows the correlation coefficient matrix of HHIs of hospital markets defined by different definition methods for 11 selected diseases. For all the selected diseases, correlation coefficients of HHIs based on different market definition methods are all significantly >0, namely, HHIs of hospital markets defined by different definition methods are significantly and positively correlated. This result suggests that the structure measurement of hospital markets defined by different methods is significantly and positively correlated.

**Table 4:** The correlation coefficient matrix of HHI of the market defined by different definition methods.

**HHI**	**HHI**				
	**Geopolitical boundaries**	**Fixed radius**	**Variable radius**	**Actual patient flow**	**Predicted patient flow**
**Non-insulin dependent diabetes (E11)**					
Geopolitical boundaries	1.000				
Fixed radius	0.531^***^	1.000			
Variable radius	0.395^***^	0.340^***^	1.000		
Actual patient flow	0.548^***^	0.125^***^	0.191^***^	1.000	
Predicted patient flow	0.489^***^	0.781^***^	0.320^***^	0.134^***^	1.000
**Schizophrenia (F20)**					
Geopolitical boundaries	1.000				
Fixed radius	0.318^***^	1.000			
Variable radius	0.307^***^	0.446^***^	1.000		
Actual patient flow	0.459^***^	0.219^***^	0.219^***^	1.000	
Predicted patient flow	0.224^***^	0.691^***^	0.393^***^	0.169**	1.000
**Senile cataract (H25–H26)**					
Geopolitical boundaries	1.000				
Fixed radius	0.440^***^	1.000			
Variable radius	0.495^***^	0.345^***^	1.000		
Actual patient flow	0.423^***^	0.382^***^	0.240^***^	1.000	
Predicted patient flow	0.354^***^	0.820^***^	0.317^***^	0.315^***^	1.000
**Chronic ischemic heart disease (I25)**					
Geopolitical boundaries	1.000				
Fixed radius	0.615^***^	1.000			
Variable radius	0.332^***^	0.354^***^	1.000		
Actual patient flow	0.677^***^	0.329^***^	0.229^***^	1.000	
Predicted patient flow	0.562^***^	0.878^***^	0.337^***^	0.293^***^	1.000
**Cerebral infarction (I63)**					
Geopolitical boundaries	1.000				
Fixed radius	0.591^***^	1.000			
Variable radius	0.332^***^	0.317^***^	1.000		
Actual patient flow	0.639^***^	0.227^***^	0.140^***^	1.000	
Predicted patient flow	0.563^***^	0.844^***^	0.312^***^	0.191^***^	1.000
**Pneumonia (J12–J18)**					
Geopolitical boundaries	1.000				
Fixed radius	0.493^***^	1.000			
Variable radius	0.327^***^	0.248^***^	1.000		
Actual patient flow	0.792^***^	0.309^***^	0.294^***^	1.000	
Predicted patient flow	0.472^***^	0.822^***^	0.215^***^	0.305^***^	1.000
**Chronic obstructive pulmonary disease (J44)**					
Geopolitical boundaries	1.000				
Fixed radius	0.688^***^	1.000			
Variable radius	0.412^***^	0.391^***^	1.000		
Actual patient flow	0.826^***^	0.456^***^	0.305^***^	1.000	
Predicted patient flow	0.654^***^	0.830^***^	0.355^***^	0.422^***^	1.000
**Cholelithiasis (K80)**					
Geopolitical boundaries	1.000				
Fixed radius	0.602^***^	1.000			
Variable radius	0.282^***^	0.265^***^	1.000		
Actual patient flow	0.511^***^	0.200^***^	0.131^***^	1.000	
Predicted patient flow	0.559^***^	0.839^***^	0.234^***^	0.209^***^	1.000
**Spinal ankyloses (M47)**					
Geopolitical boundaries	1.000				
Fixed radius	0.704^***^	1.000			
Variable radius	0.421^***^	0.373^***^	1.000		
Actual patient flow	0.736^***^	0.471^***^	0.320^***^	1.000	
Predicted patient flow	0.677^***^	0.907^***^	0.355^***^	0.447^***^	1.000
**Intervertebral disc disease (M50–M51)**					
Geopolitical boundaries	1.000				
Fixed radius	0.664^***^	1.000			
Variable radius	0.358^***^	0.346^***^	1.000		
Actual patient flow	0.711^***^	0.337^***^	0.215^***^	1.000	
Predicted patient flow	0.625^***^	0.857^***^	0.281^***^	0.321^***^	1.000
**Obstructive and reflux uropathy (N13)**					
Geopolitical boundaries	1.000				
Fixed radius	0.552^***^	1.000			
Variable radius	0.298^***^	0.270^***^	1.000		
Actual patient flow	0.586^***^	0.284^***^	0.119^***^	1.000	
Predicted patient flow	0.422^***^	0.814^***^	0.191^***^	0.213^***^	1.000

*** *p < 0.001*.

The results also show that the correlation coefficient between HHIs of markets defined by the fixed radius and predicted patient flow approach is larger than others, which means that the correlation between the structure measurement of hospital markets defined by them is greater than that of the market defined by other methods.

### Regression Analysis

**Figure 2:**
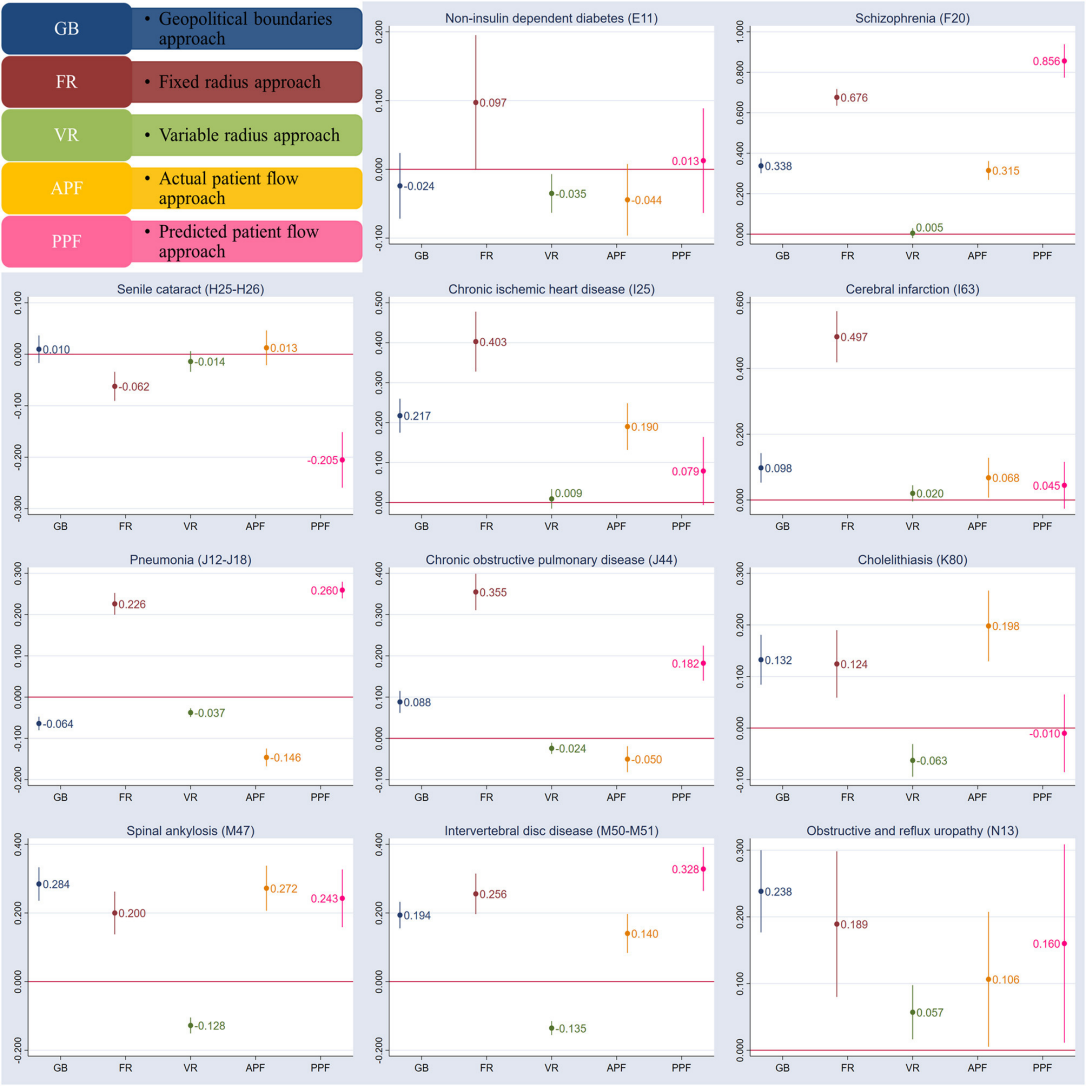
The parameter estimates of each HHI and its 95% confidence interval based on different definition method of selected diseases.

Figure 2 presents the parameter estimates of each HHI and their 95% confidence interval based on different definition methods. The results show that the coefficients of HHIs are different in identical regression models, suggesting that inferences about the effect of competition on inpatient costs would be inconsistent when alternative hospital market definition methods are employed.

Considering that the predicted patient flow has been argued by the literature of its advantages compared with the previous approaches (5, 11, 31), we used the HHI based on the predicted patient flow as a reference, and then compared the parameter estimates of HHI of the predicted patient flow with that of HHIs based on other definition methods. For all selected diseases, the parameter estimates of HHI of the market defined by fixed radius methods are consistent with that of the market defined by predicted patient flow in the direction. Combining the results of the correlation analysis, that is, the correlation coefficients between HHIs based on fixed radius and predicted patient flow approach is larger than others, we can conclude that the structure of the hospital market defined by fixed radius methods is more similar to that of the market defined by the predicted patient flow.

### Robust Test

We conducted a series of tests to verify the results of robustness.

#### Different Cut-Off

For the actual patient flow approach, we also set the exclusion threshold at 3 and 0%, respectively. County *c* was excluded from the collection of geographic areas of hospital *h* if the number of inpatients from county *c* treated in hospital *h* is <3 or 0% (i.e., no county is excluded) of the total inpatient volume of hospital *h* (16, 17). In other words, only if county *c* meets the criterion *(NP*_*ch*_*/NP*_*h*_*)* ≥ *3%* or ≥ *0%* could it be included as the elements of the geographic area collection of hospital *h*.

For the predicted patient flow approach, the choice set *J* of the patients was restricted to their chosen hospital and all hospitals within 80, 150 m, and 200 km to test the robustness of results.

For the fixed radius approach, we also employed different radii (20 and 10 miles) to test the robustness of our findings.

All of the above results are similar to the main analysis, showing the robustness of our results^9^.

#### Different Sample

**Figure 3:**
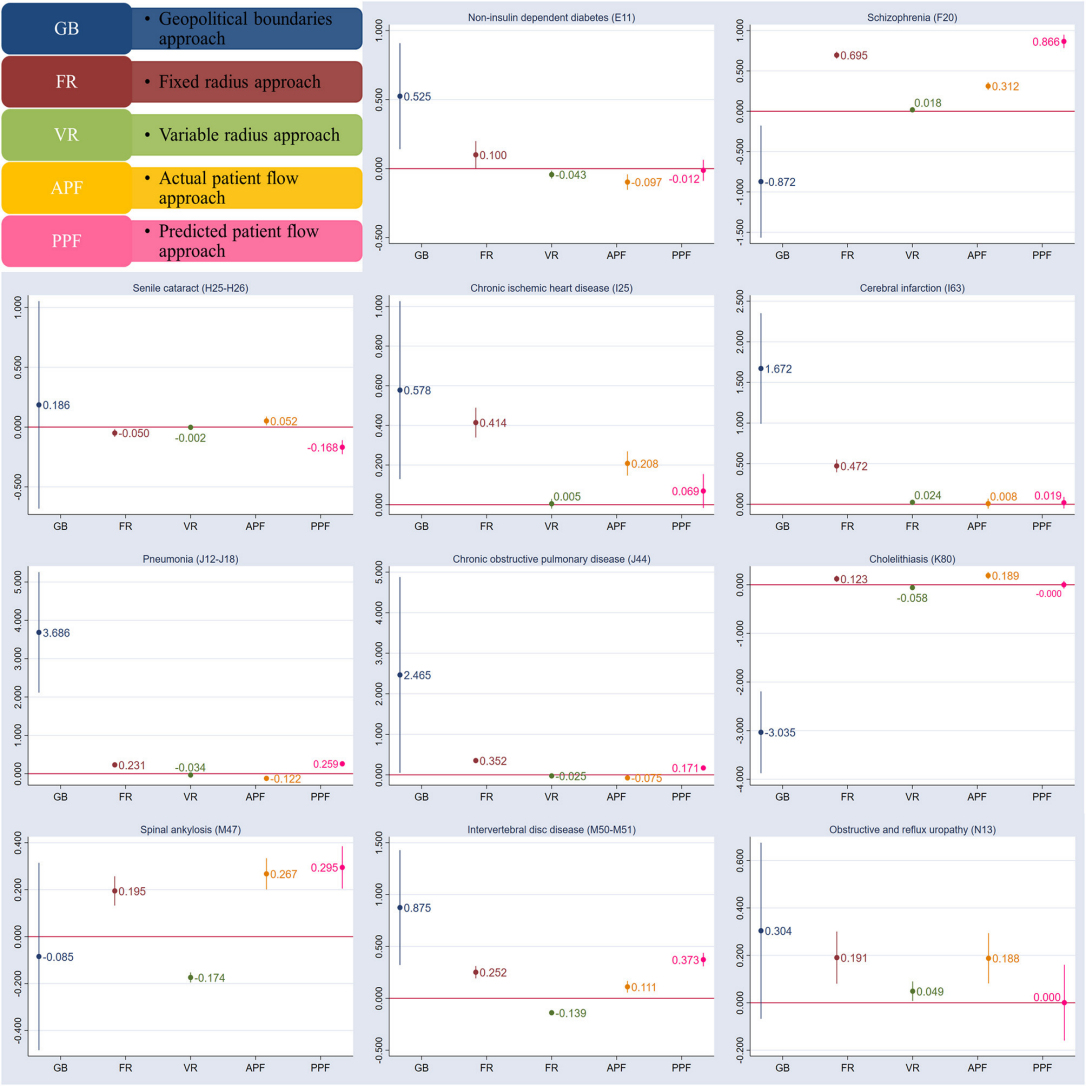
Excluding the inpatients living outside of Sichuan Province: the parameter estimates of each HHI and its 95% confidence interval.

The patient-level data used in this study were collected from the discharge data of inpatients in the fourth quarter of 2018 (from September to December) in Sichuan province. This data contains all inpatients treated in Sichuan hospitals, which means that the inpatients living outside the Sichuan province but treated in Sichuan hospitals were also included^10^. The consideration or motivation of inpatients in their selection of hospitals may be different from those living in the Sichuan province, so we excluded the inpatients living outside of Sichuan Province to verify the robustness of the results. Table 5 shows the correlation coefficient matrix of HHIs of hospital markets defined by different definition methods after excluding the inpatients living outside Sichuan Province. Figure 3 presents the parameter estimates of each HHI and their 95% confidence interval based on different definition methods after excluding the inpatients living outside Sichuan Province. The results are similar to the main analysis, showing the robustness of our results.

**Table 5:** Excluding the inpatients living outside of Sichuan Province: the correlation coefficient matrix of HHIs.

**HHI**	**HHI**				
	**Geopolitical boundaries**	**Fixed radius**	**Variable radius**	**Actual patient flow**	**Predicted patient flow**
**Non-insulin dependent diabetes (E11)**					
Geopolitical boundaries	1.000				
Fixed radius	0.344^***^	1.000			
Variable radius	0.206^***^	0.352^***^	1.000		
Actual patient flow	0.046^***^	0.324^***^	0.385^***^	1.000	
Predicted patient flow	0.253^***^	0.723^***^	0.336^***^	0.327^***^	1.000
**Schizophrenia (F20)**					
Geopolitical boundaries	1.000				
Fixed radius	0.051^***^	1.000			
Variable radius	0.046^***^	0.329^***^	1.000		
Actual patient flow	0.010**	0.283^***^	0.207^***^	1.000	
Predicted patient flow	0.024^***^	0.578^***^	0.465^***^	0.149^***^	1.000
**Senile cataract (H25–H26)**					
Geopolitical boundaries	1.000				
Fixed radius	0.269^***^	1.000			
Variable radius	0.355^***^	0.292^***^	1.000		
Actual patient flow	0.104^***^	0.454^***^	0.321^***^	1.000	
Predicted patient flow	0.230^***^	0.812^***^	0.302^***^	0.343^***^	1.000
**Chronic ischemic heart disease (I25)**					
Geopolitical boundaries	1.000				
Fixed radius	0.354^***^	1.000			
Variable radius	0.157^***^	0.400^***^	1.000		
Actual patient flow	0.095^***^	0.495^***^	0.361^***^	1.000	
Predicted patient flow	0.300^***^	0.749^***^	0.413^***^	0.425^***^	1.000
**Cerebral infarction (I63)**					
Geopolitical boundaries	1.000				
Fixed radius	0.450^***^	1.000			
Variable radius	0.199^***^	0.383^***^	1.000		
Actual patient flow	0.107^***^	0.325^***^	0.283^***^	1.000	
Predicted patient flow	0.389^***^	0.756^***^	0.373^***^	0.313^***^	1.000
**Pneumonia (J12–J18)**					
Geopolitical boundaries	1.000				
Fixed radius	0.487^***^	1.000			
Variable radius	0.201^***^	0.405^***^	1.000		
Actual patient flow	0.148^***^	0.407^***^	0.518^***^	1.000	
Predicted patient flow	0.344^***^	0.786^***^	0.435^***^	0.433^***^	1.000
**Chronic obstructive pulmonary disease (J44)**					
Geopolitical boundaries	1.000				
Fixed radius	0.387^***^	1.000			
Variable radius	0.154^***^	0.376^***^	1.000		
Actual patient flow	0.174^***^	0.476^***^	0.326^***^	1.000	
Predicted patient flow	0.473^***^	0.777^***^	0.410^***^	0.485^***^	1.000
**Cholelithiasis (K80)**					
Geopolitical boundaries	1.000				
Fixed radius	0.523^***^	1.000			
Variable radius	0.281^***^	0.395^***^	1.000		
Actual patient flow	0.156^***^	0.248^***^	0.347^***^	1.000	
Predicted patient flow	0.460^***^	0.793^***^	0.324^***^	0.207^***^	1.000
**Spinal ankylosis (M47)**					
Geopolitical boundaries	1.000				
Fixed radius	0.403^***^	1.000			
Variable radius	0.169^***^	0.332^***^	1.000		
Actual patient flow	0.205^***^	0.582^***^	0.405^***^	1.000	
Predicted patient flow	0.384^***^	0.908^***^	0.345^***^	0.574^***^	1.000
**Intervertebral disc disease (M50–M51)**					
Geopolitical boundaries	1.000				
Fixed radius	0.472^***^	1.000			
Variable radius	0.177^***^	0.373^***^	1.000		
Actual patient flow	0.196^***^	0.448^***^	0.397^***^	1.000	
Predicted patient flow	0.482^***^	0.869^***^	0.315^***^	0.377^***^	1.000
**Obstructive and reflux uropathy (N13)**					
Geopolitical boundaries	1.000				
Fixed radius	0.341^***^	1.000			
Variable radius	0.187^***^	0.379^***^	1.000		
Actual patient flow	0.063^***^	0.465^***^	0.286^***^	1.000	
Predicted patient flow	0.295^***^	0.762^***^	0.248^***^	0.337^***^	1.000

*** *p < 0.001*.

## Discussion

In this study, we selected common diseases with significant economic burden in terms of medical costs and volume of the inpatients, and employed HHI as the proxy of competition degree used as an example to measure the hospital market structure. Correlation coefficients of HHIs based on different definition methods were assessed. The corresponding coefficient of each HHI estimated in identical regression models that each HHI was independently used as an independent variable and inpatient costs as a dependent variable were then compared. We provided empirical evidence for the controversy about how big is the difference in the structure measurement of the hospital market defined by different methods, and whether the inference about the effectiveness of hospital market structure is consistent if alternative hospital market definition methods are employed.

Compared with previous studies (16, 17), this study considered the heterogeneity effects among diseases on the market definition, and the predicted patient flow was also compared with other methods. The main results show although HHIs based on different hospital market definition methods are significantly and positively correlated, the parameter estimates of them are different in the identical regression model, suggesting that the inferences about the effectiveness of market structure would be inconsistent when alternative hospital market definition methods are employed. Taking the predicted patient flow as a reference, for all selected diseases, the correlation coefficient between HHIs of the market defined by the fixed radius and predicted patient flow approach is larger than others, and their coefficient estimated in the identical regression model is consistent in the direction.

The findings of the studies about the effects of hospital market structure on hospital performance (especially for the effects of hospital competition on medical costs) are inconsistent (6, 7). One of the reasons would be that the differences in the research object and the structural variations of the research setting, such as social and economic development and healthcare system settings. For some studies using the same study object and conducted in the same healthcare delivery and regulatory environment, however, the empirical evidence on the effectiveness of hospital structure is also mixed. Combined with the findings of this study, we think that the different market definition methods employed in these studies may be one of the main reasons for the inconsistency.

The researchers should thoroughly consider the condition of the healthcare service system and the related policy regulation when selecting the market definition method to define the hospital market in the study. For example, due to the imperfect medical treatment settlement service in different places of basic health insurance^11^ before 2014, most patients could only be reimbursed for the treatments in the contract hospital located in the county where they enrolled in the basic health insurance (58, 59), leading to the patients rarely seek medical treatment outside the county where they enrolled in the basic health insurance. In this case, compared with other definition methods, the geopolitical boundaries approach is undoubtedly the most suitable choice. In 2014, the policy about the improvement of the health insurance reimbursement system for medical treatment in different places was implemented (58, 59). With the improvement of the reimbursement system for medical treatment in different places, patients who seek medical treatment outside the county where they enrolled in the basic health insurance are increasing. In addition, the transportation cost of patients has also been greatly reduced with the rapid development of high-speed rail, prompting many patients to seek medical treatment in different places. Take our data as an example, for all selected diseases, an average of 25% of the inpatients are treated in the county different from where they live. Therefore, the geopolitical boundaries approach would be not a reasonable method to define the hospital market in this condition. It is recommended that researchers should fully consider the condition of the healthcare service system and the related policy regulation to choose the appropriate definition method to define the hospital market.

Although the predicted patient flow method proposed by Kessler and Mcclellan (5) has been argued by the literature of its advantages compared with the previous approaches (5, 11, 31), it is still rarely used in the related studies due to high data requirements and complex calculation process (16). In this study, we used the HHI based on this method as a reference and compared it with others. We found that for all selected diseases, the correlation coefficients between HHIs based on fixed radius and predicted patient flow approach is larger than others, and their parameter estimates are also consistent in the direction. Based on these findings, we could infer that the structure of the hospital market defined by fixed radius methods is more similar to that of the market defined by predicted patient flow, meaning that the inference about the effectiveness of market structure would be consistent when the fixed radius method was used to replace the predicted patient flow approach in most cases. When researchers want to use the predicted patient flow method to define the hospital market, but are hindered by the data limitations and computational complexity, the fixed radius is the best alternative.

There are some limitations: (1) This study used the inpatient data of the fourth quarter of 2018. The potential seasonal trends might impact the results. In future, we would collect longitudinal data for analysis. (2) Due to the lack of the gold standard, it is impossible to determine which definition method is the best. In future, we can collect the perceived competitive pressure of hospital managers or through the simulation method to further compare the definition methods. (3) We only used the competition degree to measure the market structure; for other market structure measurements, there may be slight differences. In future, the other market structure measurement, such as the privatization rate and market size, could also be used to measure the hospital market structure and then make comparisons.

## Data Availability Statement

The data analyzed in this study is subject to the following licenses/restrictions: The data that support the findings of this study are available from the Health Commission of Sichuan Province but restrictions apply to the availability of these data, which were used under license for the current study, and so are not publicly available. Data are however available from the authors upon reasonable request and with permission of the Health Commission of Sichuan Province. Requests to access these datasets should be directed to luliyongscu@163.com.

## Ethics Statement

Ethical approval for this study and written informed consent from the participants of the study were not required in accordance with local legislation and national guidelines. This study was based on secondary data from the Sichuan Provincial Health Statistics Support System Database. All data were de-identified and the manuscript does not contain any individual person's data. Therefore, this study was exempted from ethics approval.

## Author Contributions

JP conceptualized the study. LL processed and analyzed the relevant data, as well as wrote the manuscript. TC and TL helped to process the data and analyze the relevant data. All authors contributed to the study design, interpretation the results and manuscript revision, and have approved the final manuscript.

## Conflict of Interest

The authors declare that the research was conducted in the absence of any commercial or financial relationships that could be construed as a potential conflict of interest.

## Publisher's Note

All claims expressed in this article are solely those of the authors and do not necessarily represent those of their affiliated organizations, or those of the publisher, the editors and the reviewers. Any product that may be evaluated in this article, or claim that may be made by its manufacturer, is not guaranteed or endorsed by the publisher.

## Footnotes

^1^Some excellent studies systematically review the market definition methods and competition measurements (25–28). They only introduced the principles of the definition methods and measurements. However, they did not compare whether the competition of the hospital market defined by different market definition methods is consistent.

^2^M often takes an integer between 4 and 8. For example, when M is equal to 4, it represents the concentration of the four hospitals with the largest share in the hospital market.

^3^HHI is calculated by summing the squares of the market share of each hospital usually calculated by the medical revenue of the hospital or hospital discharge. The HHI ranges from a minimum of *1/n* (when the *n* is large enough, the HHI would close to zero) to a maximum of 1, where *n* is the number of hospitals in the market with higher values representing decreased market competition (or increased market concentration). Some studies used the original HHI multiplied by 10,000 to measure the hospital market competition degree, and it ranges from close zero to 10,000 (16, 48).

^4^The market definition approaches are divided into two categories, namely theoretical and empirical definition. The theoretical definition methods include the cross-elasticity of demand and the Elzinga–Hogarty approach, and the empirical definition methods include the geopolitical boundaries, fixed radius, variable radius, actual patient flow, and predicted patient flow approach (50, 51). In most studies, the hospital market is defined by empirical definition methods rather than theoretical definition methods (6, 16, 17, 51), so this study only compared the empirical definition method.

^5^The eastern region of Sichuan is mostly covered by plains, while the central region by hills, and the western region by mountains. The per capita GDP of the most developed cities in the province of Sichuan Province (such as Panzhihua and Chengdu city) is nearly four times that of poor areas (such as Tibetan Autonomous Prefecture of Garzê and Guangyuan city). The population of Sichuan Province also consists of multiple ethnic groups, such as the Qiang people in the Aba region, the Tibetan people in the Ganzi region, and the Yi people in the Liangshan area.

^6^Most of the gynecological and obstetrics-related diseases are treated in maternity and child healthcare centers rather than hospitals within China; those diseases were excluded from our data.

^7^The descriptive statistics of the variables of each disease are shown in Appendix Table A.

^8^The reason why we chose to analyze the effects of hospital competition on inpatient costs is that this question is the most frequently studied in the empirical literature on hospital competition, and the findings are still inconsistent (6, 16, 27).

^9^Due to the space limitation, these results are not reported in the paper, but available upon request.

^10^For all selected diseases, about 1% of the inpatients living outside Sichuan Province.

^11^The basic health insurance programs include urban employee basic medical insurance, urban residence basic medical insurance, and new rural cooperative medical insurance. Currently, more than 95% of citizens in China are covered by basic health insurance (44).
