# Supplementary material for: The Comparison Between Different Hospital Market Definition Approaches: An Empirical Analysis of 11 Representative Diseases in Sichuan Province, China
Source: Front Public Health. 2021 Aug 18;9:721504. doi: 10.3389/fpubh.2021.721504 (PMC8416469; doi:10.3389/fpubh.2021.721504)
Supplement: Supplementary file 1 [file Table_1.DOCX]

**Appendix Table A: Descriptive statistics**

| Disease  Variables | E11 | F20 | H25-H26 | I25 | I63 | J12-18 | J44 | K80 | M47 | M50-M51 | N13 |
| --- | --- | --- | --- | --- | --- | --- | --- | --- | --- | --- | --- |
| ***Inpatient costs*** | 7,771.45  (8,226.37) | 15,094.19  (13,570.14) | 59,99.96  (3,376.13) | 8,168.21  (11,548.00) | 9,869.59  (12,913.69) | 5,193.81  (10,876.40) | 7,515.83  (7,699.11) | 10,215.51  (8,476.46) | 4,710.59  (4,856.60) | 5,880.38  (7,810.23) | 7,286.35  (7,976.68) |
| ***HHI-GB*** | 0.36  (0.17) | 0.82  (0.21) | 0.44  (0.20) | 0.28  (0.13) | 0.31  (0.14) | 0.44  (0.22) | 0.24  (0.14) | 0.39  (0.16) | 0.19  (0.13) | 0.20  (0.13) | 0.38  (0.18) |
| ***HHI-FR*** | 0.10  (0.10) | 0.25  (0.21) | 0.15  (0.14) | 0.07  (0.07) | 0.08  (0.07) | 0.12  (0.14) | 0.06  (0.09) | 0.10  (0.12) | 0.05  (0.08) | 0.05  (0.08) | 0.10  (0.11) |
| ***HHI-VR*** | 0.19  (0.24) | 0.28  (0.31) | 0.15  (0.21) | 0.17  (0.21) | 0.17  (0.21) | 0.26  (0.29) | 0.16  (0.23) | 0.18  (0.23) | 0.14  (0.20) | 0.13  (0.20) | 0.17  (0.24) |
| ***HHI-APF*** | 0.24  (0.12) | 0.60  (0.18) | 0.27  (0.12) | 0.20  (0.10) | 0.22  (0.10) | 0.30  (0.16) | 0.19  (0.11) | 0.24  (0.09) | 0.15  (0.09) | 0.15  (0.08) | 0.23  (0.10) |
| ***HHI-PPF*** | 0.06  (0.82) | 0.17  (0.11) | 0.07  (0.08) | 0.06  (0.07) | 0.07  (0.08) | 0.11  (0.17) | 0.06  (0.09) | 0.06  (0.11) | 0.04  (0.06) | 0.04  (0.07) | 0.04  (0.07) |
| ***Sex*** |  |  |  |  |  |  |  |  |  |  |  |
| Male | 21,800  (50.97) | 30,777  (65.45) | 31,988  (44.49) | 35,486  (46.57) | 42,769  (53.86) | 97,459  (54.26) | 101,115  (66.71) | 19,553  (33.06) | 21,633  (36.65) | 43,530  (43.63) | 24,078  (66.25) |
| Female | 20,968  (49.03) | 16,245  (34.55) | 39,819  (55.38) | 40,710  (53.43) | 36,641  (46.14) | 82,147  (45.73) | 50,442  (33.28) | 39,584  (66.93) | 37,391  (63.34) | 56,230  (56.36) | 12,266  (33.75) |
| Missing | 2  (0.00) | 0  (0.00) | 98  (0.14) | 0  (0.00) | 3  (0.00) | 13  (0.01) | 10  (0.01) | 2  (0.00) | 4  (0.01) | 4  (0.00) | 0  (0.00) |
| ***Age*** | 62.90  (12.97) | 47.18  (13.73) | 70.22  (9.31) | 72.54  (10.69) | 70.67  (11.23) | 19.78  (28.99) | 72.88  (9.75) | 54.09  (15.95) | 55.92  (13.53) | 57.83  (14.09) | 48.75  (14.57) |
| ***Health insurance*** |  |  |  |  |  |  |  |  |  |  |  |
| URBMI | 15,789  (36.92) | 27,403  (58.28) | 38,042  (52.91) | 35,523  (46.62) | 37,774  (47.57) | 88,773  (49.42) | 70,341  (46.41) | 25,278  (42.74) | 25,068  (42.47) | 42,548  (42.65) | 163,25  (44.92) |
| UEBMI | 16,478  (38.53) | 5,097  (10.84) | 13,864  (19.28) | 21,690  (28.47) | 20,532  (25.85) | 22,486  (12.52) | 40,827  (26.94) | 13,702  (23.17) | 22,641  (38.36) | 31,848  (31.92) | 8,048  (22.14) |
| NCMS | 4,827  (11.29) | 9,361  (19.91) | 10,239  (14.24) | 9,596  (12.59) | 10,793  (13.59) | 27,017  (15.04) | 22,763  (15.02) | 10,223  (17.29) | 6,613  (11.20) | 14,865  (14.90) | 6,036  (16.61) |
| Full self-expenses | 25,57  (5.98) | 1,526  (3.25) | 3,619  (5.03) | 3,106  (4.08) | 4,275  (5.38) | 25,384  (14.13) | 5,397  (3.56) | 4,924  (8.33) | 1,914  (3.24) | 3,775  (3.78) | 3,455  (9.51) |
| Other | 3,119  (7.29) | 3,635  (7.73) | 6,141  (8.54) | 6,281  (8.24) | 6,039  (7.60) | 15,959  (8.88) | 12,239  (8.07) | 5,012  (8.47) | 2,792  (4.73) | 6,728  (6.74) | 2,480  (6.82) |
| ***Admission source*** |  |  |  |  |  |  |  |  |  |  |  |
| Emergency department | 7,000  (16.37) | 3,854  (8.20) | 4,536  (6.31) | 13,171  (17.29) | 22,099  (27.83) | 37,693  (20.98) | 26,491  (17.48) | 15,808  (26.73) | 3,595  (6.09) | 8,244  (8.26) | 9,541  (26.25) |
| Others department | 35,770  (83.63) | 43,168  (91.80) | 67,369  (93.69) | 63,025  (82.71) | 57,314  (72.17) | 141,926  (79.02) | 125,076  (82.52) | 43,331  (73.27) | 55,433  (93.91) | 91,520  (91.74) | 26,803  (73.75) |
| ***Urgency when admission*** |  |  |  |  |  |  |  |  |  |  |  |
| Critical urgent or Urgent | 10,070  (23.54) | 3,406  (7.24) | 734  (1.02) | 26,884  (35.28) | 32,138  (40.47) | 56,853  (31.65) | 56,368  (37.19) | 13,608  (23.01) | 5,321  (9.01) | 83,35  (8.35) | 8,183  (22.52) |
| Common | 32,700  (76.46) | 43,616  (92.76) | 71,171  (98.98) | 49,312  (64.72) | 47,275  (59.53) | 122,766  (68.35) | 95,199  (62.81) | 45,531  (76.99) | 53,707  (90.99) | 91,429  (91.65) | 28,161  (77.48) |
| ***Number of secondary diagnosis*** | 5.60  (3.36) | 0.99  (1.57) | 1.28  (1.43) | 1.94  (1.72) | 4.69  (2.86) | 1.55  (2.56) | 4.42  (2.91) | 2.12  (2.20) | 2.25  (2.06) | 2.23  (2.12) | 1.87  (1,91) |
| ***CCI*** | 3.46  (3.06) | 0.23  (0.65) | 0.22  (0.66) | 0.28  (0.13) | 1.49  (1.56) | 0.47  (1.22) | 1.60  (1.50) | 0.76  (1.28) | 0.51  (0.86) | 0.39  (0.80) | 0.32  (0.81) |
| ***Whether general*** |  |  |  |  |  |  |  |  |  |  |  |
| No | 11,366 (26.57) | 36,543 (77.71) | 34,773 (48.36) | 17,199 (22.57) | 19,829  (24.97) | 38,747  (21.57) | 33,684  (22.22) | 11,018  (18.63) | 23,775  (40.28) | 43,517  (43.62) | 7,346  (20.21) |
| Yes | 31,404  (73.43) | 10,479  (22.29) | 37,132  (51.64) | 58,997  (77.43) | 59,584  (75.03) | 140,872  (78.43) | 117,883  (77.78) | 48,121  (81.37) | 35,253  (59.72) | 56,247  (56.38) | 28,998  (79.79) |
| ***Hospital level*** |  |  |  |  |  |  |  |  |  |  |  |
| Primary | 641  (1.50) | 1,345  (2.86) | 2,473  (3.44) | 4,008  (5.26) | 2,314  (2.91) | 3,103  (1.73) | 8,778  (5.79) | 1,177  (1.99) | 6,856  (11.61) | 8,562  (8.58) | 2,706  (7.45) |
| Secondary | 10,577 (24.73) | 20,245  (43.05) | 18,594  (25.86) | 23,614  (30.99) | 27,052  (34.06) | 55,308  (30.79) | 51,798  (34.17) | 17,894  (30.26) | 18,757  (31.78) | 36,330  (36.42) | 11,525  (31.71) |
| Tertiary | 27,510 (64.32) | 10,819  (23.01) | 30,867  (42.93) | 37,340  (49.01) | 41,968  (52.85) | 109,192  (60.79) | 61,092  (40.31) | 36,543  (61.79) | 13,894  (23.54) | 27,086  (27.15) | 18,517  (50.95) |
| Un-graded | 4,042  (9.45) | 14,613  (31.08) | 19,971  (27.77) | 11,234  (14.74) | 8,079  (10.17) | 12,016  (6.69) | 29,899  (19.73) | 3,525  (5.96) | 19,521  (33.07) | 27,786  (27.85) | 3,596  (9.89) |
| ***Whether for-profit*** |  |  |  |  |  |  |  |  |  |  |  |
| No | 38,085  (89.05) | 39,945  (84.95) | 41,610  (57.87) | 62,451  (81.96) | 68,017  (85.65) | 166,209  (92.53) | 118,714  (78.32) | 52,721  (89.15) | 38,317  (64.91) | 66,893  (67.05) | 29,448  (81.03) |
| Yes | 4,685  (10.95) | 7,077  (15.05) | 30,295  (42.13) | 13,745  (18.04) | 11,396  (14.35) | 13,410  (7.47) | 32,853  (21.68) | 6,418  (10.85) | 20,711  (35.09) | 32,871  (32.95) | 6,896  (18.97) |
| ***Hospital ownership*** |  |  |  |  |  |  |  |  |  |  |  |
| Public | 35,703  (83.48) | 32,134  (68.34) | 36,883  (51.29) | 54,519 (71.55) | 61,643 (77.62) | 156,609 (87.19) | 101,157 (66.74) | 49,690 (84.02) | 27,209 (46.10) | 51,002 (51.12) | 25,572  (70.36) |
| Private | 7,067  (16.52) | 14,888  (31.66) | 35,022  (48.71) | 21,677 (28.45) | 17,770 (22.38) | 23,010 (12.81) | 50,410  (33.26) | 9,449  (15.98) | 31,819  (53.90) | 48,762  (48.88) | 10,772  (29.64) |
| ***Health Personnel*** | 9.66  (4.97) | 7.21  (3.76) | 9.90  (5.34) | 8.17  (4.72) | 8.36  (4.65) | 9.09  (5.25) | 7.64  (4.06) | 9.02  (5.12) | 8.10  (4,31) | 8.10  (4.61) | 8.62  (4.54) |
| ***GDP*** | 66,674.28  (34,509.34) | 49,662.97  (24,852.43) | 64,575.89  (33,502.73) | 56,020.07  (32,015.53) | 56,484.65  (31,168.73) | 63,758.65  (35,661.09) | 57,353.11  (31,455.55) | 62,050.83  (33,048.35) | 58,204.54  (29,706.65) | 56,941.81  (30,938.63) | 62,698.77  (32,676.56) |
| ***Population*** | 77.73  (35.81) | 70.34  (28.44) | 82.60  (36.25) | 74.23  (34.50) | 72.49  (33.02) | 72.12  (39.24) | 72.83  (33.92) | 76.78  (39.93) | 72.53  (36.05) | 72.42  (36.42) | 73.94  (37.36) |
| ***Urbanization rate (%)*** | 65.92  (21.98) | 54.00  (16.58) | 66.37  (22.59) | 58.85  (21.15) | 59.92  (20.82) | 61.75  (22.89) | 57.71  (20.45) | 61.89  (21.66) | 58.91  (21.00) | 58.13  (20.99) | 61.87  (21.42) |

Note: (1) For continuous variables, statistics shown are the sample mean and standard deviation (in parentheses). For categorical variables, statistics shown are the frequency and percentage (in parentheses) (2) HHI-GB, HHI-FR, HHI-VR, HHI-APF, HHI-PPF: the Herfindahl-Hirschman Index of the hospital market defined by geopolitical boundaries, fixed radius, variable radius, actual patient flow, predicted patient flow approach, respectively. (3) URBMI: Urban Employment Basic Medical Insurance. UEBMI: Urban Residents Basic Medical Insurance. NCMS: New Cooperative Medical Scheme. (4) CCI: Charlson comorbidity index. (5) Health personnel: the number of health personnel per 1,000 population. GDP: GDP per capita (Yuan). Population: the number of population (10,000 people). Urbanization rate: the proportion of the urban population (urbanization rate).
